# The Effect of Statins on Clinical Outcome Among Hospitalized Patients With COVID-19: A Multi-Centric Cohort Study

**DOI:** 10.3389/fphar.2022.742273

**Published:** 2022-07-05

**Authors:** Srikanth Umakanthan, Sanjum Senthil, Stanley John, Mahesh K. Madhavan, Jessica Das, Sonal Patil, Raghunath Rameshwaram, Ananya Cintham, Venkatesh Subramaniam, Madhusudan Yogi, Abhishek Bansal, Sumesh Achutham, Chandini Shekar, Vijay Murthy, Robbin Selvaraj

**Affiliations:** ^1^ Department of Para-clinical sciences, Faculty of Medical Sciences, The University of the West Indies, St Augustine, Trinidad, Trinidad and Tobago; ^2^ Project Lead and Research Programmee Committee Member, International Research Association Unit, India; ^3^ Director and Consultant in Emergency Medicine, Department of Medicine, RRN Multispeciality Hospital, India; ^4^ Consultant Pulmonologist, Department of Medicine, Holy Cross Hospital, Nagercoil, India; ^5^ National Regional Collaboration for Medical Research Foundation, India; ^6^ Department of Biostatistics, Epidemiology, and Informatics, Piramal Research Centre, Gujarat, India; ^7^ Medical Residents, Swaminathan Multispecialty Hospital, Chennai, India

**Keywords:** COVID-19, statins, cohort, mortality, propensity

## Abstract

The coronavirus disease-2019 (COVID-19) is caused by SARS-CoV-2, leading to acute respiratory distress syndrome (ARDS), thrombotic complications, and myocardial injury. Statins, prescribed for lipid reduction, have anti-inflammatory, anti-thrombotic, and immunomodulatory properties and are associated with reduced mortality rates in COVID-19 patients. Our goal was to investigate the beneficial effects of statins in hospitalized COVID-19 patients admitted to three multi-specialty hospitals in India from 1 June 2020, to 30 April 2021. This retrospective study included 1,626 patients, of which 524 (32.2%) were antecedent statin users among 768 patients (384 statin users, 384 non-statin users) identified with 1:1 propensity-score matching. We established a multivariable logistic regression model to identify the patients’ demographics and adjust the baseline clinical and laboratory characteristics and co-morbidities. Statin users showed a lower mean of white blood cell count (7.6 × 10^3^/µL vs. 8.1 × 10^3^/µL, *p* < 0.01), and C-reactive protein (100 mg/L vs. 120.7 mg/L, *p* < 0.001) compared to non-statin COVID-19 patients. The same positive results followed in lipid profiles for patients on statins. Cox proportional-hazards regression models evaluated the association between statin use and mortality rate. The primary endpoint involved mortality during the hospital stay. Statin use was associated with lower odds of mortality in the propensity-matched cohort (OR 0.52, 95% CI 0.33-0.64, *p* < 0.001). These results support the previous evidence of the beneficial effects of statins in reducing mortality in hospitalized COVID-19 patients.

## Introduction

The coronavirus disease (COVID-19) derives from the novel coronavirus SARS-CoV-2, which has caused a global pandemic since March 2020 (Umakanthan et al., 2020). The hospitalized patients suffering from COVID-19 have presented with a range of clinical manifestations that include mild respiratory illness to severe respiratory failure (Parasher, 2021).

Based on the categorical classification of severity of illness and complications, the management of COVID-19 has ranged from intubation to mechanical ventilation combating acute respiratory distress syndrome (ARDS) (Umakanthan et al., 2022). The initial phase of COVID-19 management depended on the patient’s investigational outcome of lung function test, pulmonary vascular resistance, and vascular endothelial function (Huertas et al., 2020; Potus et al., 2020). COVID-19 patients also exhibited a higher risk of systemic complications that include cardiovascular system (myocardial infarction, myocarditis), cerebrovascular events (hemorrhagic infarctions, encephalopathy), and thromboembolic events. The COVID-19 patients’ often presented with significant co-morbidities that included diabetes, hypertension, obesity, and a previous episode of ischemic heart disease (Umakanthan and Lawrence, 2022). A retrospective study conducted in China, revealed that out of 173 severe COVID-19 patients, the prevalence of hypertension, diabetes mellitus, coronary vascular disease, and cerebrovascular disease was significantly higher than non-severe diseases (Guan et al., 2020). The cholesterol-lowering drugs, statins, have shown to decrease the risk of atherosclerotic induced complications such as ischemic heart disease and its related complications (Feingold et al., 2000). Statins act in various modes that include antithrombotic, anti-inflammatory, and enhanced endothelial function (Pinal-Fernandez et al., 2018). These effects can prove beneficial in reducing mortality in COVID-19 patients, as shown in a retrospective study conducted in China (Zhang et al., 2020). Statins exhibit their cholesterol-lowering effect by inhibiting the mevalonate pathway and reduces lipid levels and enhances vascular endothelial function, causing a significant reduction in mortality due to complications arising from coronary artery disease (Zhou and Liao, 2009). The molecular pathogenesis for statins include inhibiting miR-133a expression, decreasing C-reactive protein levels (CRP), interfering with Kruppel-like factor-2 signaling, and modulating high mobility group box 1/toll-like receptor 4(HMGB1/TLR4) pathway (Gu et al., 2020).

Given the antagonist mentioned above effects of COVID-19 on the immune-inflammatory status of the host and the agonist action of statins in these patients, we performed a retrospective cohort study to investigate the effects of statins in COVID-19 hospitalized patients’ In India. The present study is the first type to be conducted on an Indian population, and it highlights the salient beneficial effects of statins in a cohort group.

## Materials and Methods

### Data Sources

In India, all citizens are assigned a unique identification number using a biometric ID system at an individual level (Unique Identification Authority of India, 2009). For this study, we collected data from three multispecialty hospitals in India. The patients’ data was verified using the unique identity number. Then, the patients’ demographic details and other relevant clinical and laboratory details were obtained from the medical records department. A superior level of confidentiality and electronically secured data storage systems were utilized throughout the study.

### Study Design and Population

This retrospective cohort study involved COVID-19 patients who were hospitalized and treated in three multispecialty hospitals in India from the time interval of 1 June 2020, to 30 April 2021.

### Inclusion and Exclusion Criteria

The inclusion criteria for the study population were COVID-19 patients hospitalized, discharged, or died during the period mentioned above. The confirmation of COVID-19 was based on positive results obtained from the SARS-CoV-2 polymerase chain reaction (PCR) test of respiratory (nasopharynx, oropharynx) specimens and oxygenation saturation (SpO2) of ≤93% or PaO2/FiO2 < 300 mmHg. Imaging studies (Chest computed tomography) supporting PCR test results were included. These testing were all conducted in the hospitals mentioned above. Patients below the age of 18 and above 80 years, those without proper medical records documentation, patients admitted for only 1 day, and those suffering from *tuberculosis*, hepatitis B, HIV, and carcinomas requiring immunomodulatory therapies were excluded. The initial case-finding tally of 1834 patients was identified and reviewed based on the inclusion criteria. Of these patients, following this scrutiny, a total of 1,626 patients were identified for our study.

### Data Extraction

The patient data was identified using an electronic data search at the medical records department in three hospitals. No manual abstraction was performed due to COVID-19 restrictions. Patient demographics and clinical data were filled in using the most commonly occurring clinical signs and symptoms. The same selection format was followed to collect laboratory results. Treatment history and clinical outcome were followed until the end of the study period, patients’ death in-hospital, or until the patients’ discharge. The data was stored using alphanumerical code, and the patient’s name was de-identified.

### Intervention

Baseline information, including age, gender, body mass index (BMI), and co-morbidities, were recorded. Clinical co-morbidities included hypertension, diabetes mellitus, coronary artery disease, chronic lung disease, chronic kidney disease, stroke, and heart failure. We included features of clinical presentations during the patient’s hospitalization (i.e., presence of fever, dyspnea, cough, chest pain, fatigue, and O2 saturation). Several laboratory parameters at the time of hospital admission were also collected from the electronic medical records, including white blood count (WBC), D-dimer, C-reactive protein (CRP), erythrocyte sedimentation rate (ESR), total cholesterol, low-density lipoprotein (LDL), high-density lipoprotein (HDL) and triglycerides. As statins act by lowering lipid levels, we collected lipid values from COVID-19 patients from inpatient and outpatient medical records and then averaged them for each patient throughout the study. Some patients failed to present the laboratory values collected during their clinical checkups; the data was validated by involving patients in whom these were available. The details for missing laboratory values are provided in Table 1. In this study population, 524 patients (32.2%) were on statin therapy, and the remaining 1,102 (67.7%) were not on statins. Among 768 patients, a 1:1 propensity-score matching (384 statin users and 384 non-statin users) was done. The comparison between statin and non-statin patients was further categorized. A detailed medical record search for individual patient demographics, clinical features, COVID-19 disease, duration of statin use, and underlying co-morbidities was elicited either from medical records or from the patients/family members and then documented. The data was reviewed by the treating physicians, pulmonologists, and radiologists to exclude any data inaccuracies.

**TABLE 1:** Missing laboratory markers in the propensity-matched cohort.

Laboratory marker	Statin use (384)	Non-statin use (384)
WBC	1 (0.2%)	4 (1%)
D-dimer	92 (23.9%)	111 (28.9%)
CRP	38 (9.8%)	43 (11.1%)
ESR	80 (20.8%)	74 (19.2%)
Total cholesterol	34 (8.8%)	47 (12.2%)
LDL	38 (9.8%)	59 (15.3%)
HDL	38 (9.8%)	59 (15.3%)
Triglycerides	5 (1.3%)	11 (2.8%)

WBC, white blood cells; CRP, C-reactive protein; ESR, erythrocyte sedimentation rates; LDL, low density lipoprotein; HDL, high density lipoprotein.

### Patient and Public Involvement

None of the patients and the public were involved during the design, data analysis, and manuscript preparation.

### Ethics Approval

The Cross-Hospital Ethics Review Board approved this study, and the waiver was granted for informed consent as patient data were de-identified.

### Statin Exposure and Study Outcomes

Antecedent statin use in this cohort study was defined as “medical data record with statin being prescribed by the patient’s physician as a routine home medication due to their previous history of hypertension, coronary artery disease, and stroke.” The statin in these patients was appeased from the patient or the family members, dependents, and pharmacies at the time of hospitalization. The primary endpoint was defined as “mortality during a hospital stay,” and the secondary endpoint was defined as “invasive mechanical ventilation during a hospital stay.” Our study outcome also included the duration of hospital stay, number of days on a ventilator, and use of hemodialysis.

### Statistical Analyses

This study’s characteristic variables included the basic demographics, associated co-morbidities, antecedent statin use, common clinical and laboratory findings, and clinical outcomes. The statistical estimates were previewed as number (percentage) [n (%)] for nominal variables, medians, and inter-quantile ranges (IQR) for measuring variables. The differences between this study’s unmatched and matched cohorts were examined using the two-sided independent *t*-test and the Chi-square test.

We constructed Cox proportional-hazard regression models to estimate the association between statin, use, and primary endpoint. Before building the Cox model, the Schoenfeld residual analysis test detected the proportional hazard (PH) for all confounders. The PH means “the ratio of the hazards for any two variables is constant over time."

**FIGURE 1:**
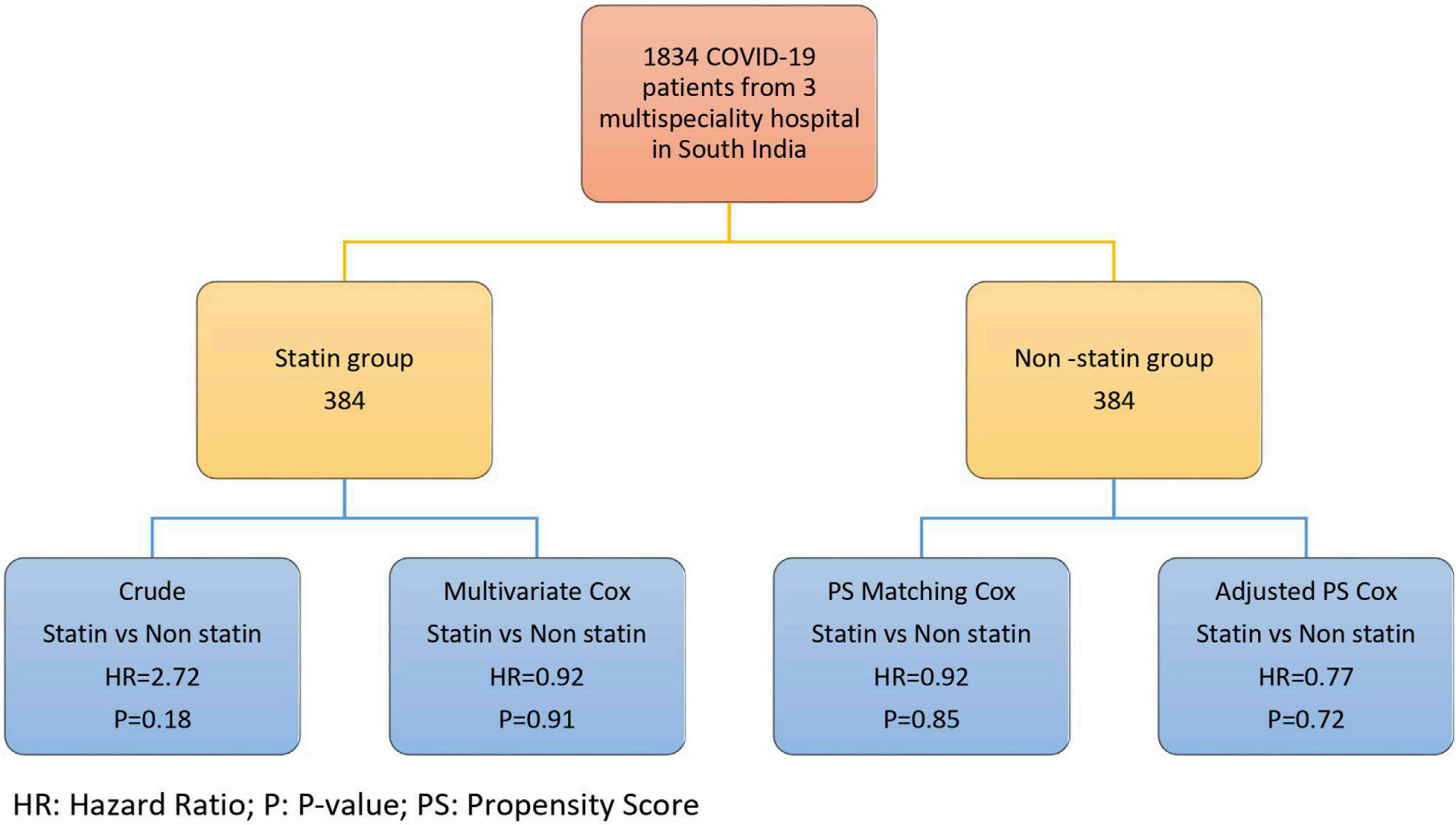
Flow-chart depicting study population and Cox proportional-hazards regression scores.

The univariable logistic regression was performed for each confounding factor. The variables that had a significant association with the mortality hazard were probed into the multivariate Cox regression. To adjust the bias resulting from the confounders, we implemented four strategies; multivariate Cox regression, weighted PH Cox, PH Cox after the propensity score matching, and the PH Cox adjusted with propensity score (Figure 1). In our study, individual matching was impossible due to the variations in the drug types used in COVID-19 therapy and other confounding variables, including the clinical and laboratory characteristics.

A confounder is a significant factor in causing variance and bias in an observational study. Due to the absence of randomization of patients in the treatment category, the cohort patients are not identical in their clinical, laboratory characteristics, and treatment strategy. To minimize the effects of the confounding factor, we implemented propensity score methods. The propensity score is a statistical analysis to estimate treatment’s pure effect and reduce or eliminate the bias caused by other confounding variables. All confounding variables’ effects were summarized into a scoring pattern, referred to as the “propensity score.” The individual propensities were estimated using the multivariate logistic regression model that included all the confounding covariates, mixed Cox model, Cox model with time-varying exposure, and marginal-structural model.

We formulated a multivariable logistic regression model to determine the confounding factors. The patients with statin index treatment were considered a dependent variable, and the baseline covariates were considered an independent variable. The propensity score matching was implemented using a nearest-neighbor strategy with the specification of caliper width equal to 0.1 of the standard deviation of the logit propensity score. All the baseline variables in the propensity-matched cohort were evaluated by implementing descriptive analysis. Laboratory results and patient clinical outcomes were stratified based on statin use in the cohort group of hospitalized COVID-19 patients.

We constructed a logistic regression on the propensity-matched cohort with the control group as a reference to address the primary and secondary endpoints. We analyzed and examined if the effect estimate remained consistent by utilizing logistic regression with multivariable adjustment on the total cohort. We modified the multivariable models for variables that have been previously studied concerning COVID-19 mortality, including the baseline covariates. Statistical package for social science (SPSS) software 2.0 was used for statistical analyses (Arbogast et al., 2013; Ranganathan et al., 2017).

### Sensitivity Analyses

Sensitivity analyses were performed by defining the recent statin users as “either antecedent statin or in-hospital statin use.” We analyzed the data by defining statin users as “in-hospital statin users.” By implementing these amended definitions, we estimated the relation of any recent statin users with the primary endpoint using multivariable logistic regression. We also utilized the subgroup analyses to evaluate the association of antecedent statin use with the primary endpoint in a subset of patients where statins are generally prescribed (e.g., history of hypertension, coronary artery disease, and stroke).

### Missing Data

Data search revealed missing BMI and prior statin use in 19 and 15% of the registered patients. We utilized multiple imputations with predictive mean matching to adjust the missing data entry variables. We imputed 100 datasets, estimated the odds ratios on each imputed data variable, and averaged the one hundred estimated values to acquire the pooled estimates. Rubin’s rules were implemented to calculate the model estimates and the standard errors (Lu et al., 2008). The patients’ co-morbidities and lipid results were missing in 18 and 55% of the cohort, and these were presented only at baseline. The remaining variables were missing in less than 5% of the cohort study.

## Results

### General Patient Characteristics

Out of 1,626 patients included in our study, 524 (32.2%) had a history of antecedent statin use before hospital admission. Based on the average age of the patients, statin users (median age of 63 years with IQR 55-79) were older in comparison to non-statin users (median age of 59 years with IQR 45-77) (*p* < 0.001). COVID-19 was marginally more in males (55.84%) and in statin users (52.4%) (*p* = 0.05) (Table 2).

**TABLE 2:** Characteristic baseline demographics and clinical manifestations in unmatched and matched (propensity) cohorts.

	Unmatched			Matched		
Total *N*= 1626	Statin use 524 (32.2%)	Non-statin use 1102 (67.7%)	*p* value	Statin use (*n*=384)	Non-statin use (*n*=384)	*p* value
Demographics Age (years)	63 (55-79)	59 (45-77)	<0.001	62 (54-76)	64 (55-77)	0.16
BMI (kg/m^2^)	28.2 (24.1-31.8)	27.8(24.4-32.0)	0.24	28.3 (24.6-32.4)	27.2 (23.8-31.8)	0.62
Gender						
Male	275 (52.4%)	633 (57.4%)	0.05	211 (54.9%)	223 (58.0%)	1.0
Female	249 (47.5%)	469 (42.5%)		173 (45.0%)	161 (41.9%)	
Co-morbidities						
HTN	393 (75.0%)	640 (58.0%)	<0.001	242 (63.0%)	273 (71.0%)	0.45
DM	328 (62.5%)	445 (40.3%)	<0.001	212 (55.2%)	224 (58.3%)	0.54
CAD	249 (47.5%)	409 (37.1%)	<0.001	62 (16.1%)	58 (15.1%)	0.7
CLD	205 (39.1%)	268 (24.3%)	<0.001	93 (24.2%)	96 (25.0%)	0.45
CKD	195 (37.2%)	176 (15.9%)	<0.01	84 (21.8%)	80 (20.8%)	0.75
CVA	58 (11%)	33 (2.9%)	<0.01	35 (9.1%)	33 (8.5%)	0.86
Heart failure	220 (41.9%)	364 (33.0%)	0.96	64 (16.6%)	62 (16.1%)	0.88
Liver disease	36 (6.8%)	44 (3.9%)	0.98	36 (9.3%)	28 (7.2%)	0.70
Clinical presentations						
Fever (^o^c)	37.45 ± 1.16	37.41 ± 1.04	0.787	37.31 ± 1.09	37.22 ± 1.04	0.60
Dyspnoea n (%)	502 (95.8%)	860 (78.0%)	<0.01	320 (83.3%)	312 (81.2%)	0.88
Cough n (%)	489 (93.3%)	901 (81.7%)	<0.01	303 (78.9%)	297 (77.3%)	0.87
Chest pain n(%)	26 (4.9%)	54 (4.9%)	<0.001	35 (9.1%)	39 (10.1%)	0.88
Fatigue n (%)	486 (92.7%)	843 (76.4%)	<0.01	296 (77.0%)	301 (78.3%)	0.86
O_2_ saturation (SpO2_2_ ≤93%)	157 (29.9%)	332 (30.1%)	<0.001	114 (29.6%)	108 (28.1%)	0.88

BMI, body mass index; HTN, hypertension; DM, diabetes mellitus; CAD, coronary artery disease; CLD, chronic lung disease; CKD, chronic kidney disease; CVA, cerebrovascular accident.

### Prevalence of Co-morbid Variables

Patients using statins had a significant higher prevalence of co-morbidities such as hypertension (75.0 vs. 58.0%), diabetes mellitus (62.5 vs. 40.3%), coronary artery disease (47.5 vs. 37.1%), and chronic lung disease (39.1 vs. 24.3%) in comparison to patients without statin intake (*p* < 0.001 for all). Furthermore, patients using statins were more likely to experience chronic kidney disease (37.2 vs. 15.9%), cerebrovascular accidents (11.0 vs. 2.9%) (*p* < 0.01 for both) (Table 2).

### Variation in the Incidences of Clinical Manifestations

At the time of initial clinical presentation, patients on statins had a high incidence of dyspnea, cough, and fatigue (95.8, 93.3, and 92.7%, respectively) in comparison to non-statin patients (78.0, 81.7, and 76.4% respectively. There were no notable differences in the patients’ presentation with chest pain and low O2 saturation among statin and non-statin users (Table 2).

### Variations in Laboratory Findings Based on Propensity-Matched Cohort Incidences Were Compared During Admission and Discharge in Statin and Non-Statin Users

At the time of discharge the laboratory results revealed that the patients with statins showed a lower mean of WBC count (7.6 × 10^3^/µL vs. 8.1 × 10^3^/µL, *p* < 0.01), and C-reactive protein (100 mg/L vs. 120.7 mg/L, *p* < 0.001) compared to non-statin COVID-19 patients. The same positive results followed in lipid profiles for patients on statins (Table 3). Patients with antecedent statin use showed no significant differences for mechanical ventilation (20 vs. 24.2%, *p* 0.07), and hemodialysis (5.4 vs. 7%, *p* 0.41), and in length of hospital stay and days on a ventilator (Table 4).

**TABLE 3:** Laboratory values in propensity-matched cohorts.

	At admission		At discharge		
Laboratory values	Statin use	Non-statin use	Statin use	Non- statin use	*p* value
*WBC count* (*10* ^3^ */µL*)	7.9 (5.5–11.4)	8.6 (5.4–12.0)	7.6 (5.2–11.1)	8.1 (5.6–11.6)	<0.01
*D-dimer (µg/ml)*	2.3 (1.3–4.1)	2.8 (1.4–5.2)	2.1 (1.1–3.8)	2.5 (1.2–4.8)	0.37
*CRP (mg/ml)*	106 (50–178)	127.6 (82.2–198.8)	100.0 (48.1–172.2)	120.7 (72.2–196.6)	<0.001
*ESR (mm/hr)*	72 (41–103)	79.8 (38.6–98.2)	67.5 (36.3–98.1)	66.5 (34.4–93.6)	0.84
*Total cholesterol (mg/dl)*	161.1 (122.2–191.1)	168.8 (137.4–206.6)	154.2 (118.3–186.0)	164.6 (133.0–203.7)	<0.01
*LDL (mg/dl)*	81.2 (61.2–112.4)	93.4 (67.4–114.4)	79.1 (58.0–110.2)	92.0 (68.0–115.0)	<0.01
*HDL (mg/dl)*	42.0 (36.2–56.4)	38.2 (32.6–46.4)	44.0 (34.4–56.4)	40.0 (32.2–53.3)	0.25
*Triglycerides (mg/dl)*	148.2 (102.4–198.4)	156.4 (102.4–212.6)	144.0 (99.2–192.3)	158.0 (94.7–216.7)	0.25

WBC, white blood cells; CRP, C-reactive protein; ESR, erythrocyte sedimentation rates; LDL, low density lipoprotein; HDL, high density lipoprotein*.*

**TABLE 4:** Clinical outcome in propensity matched cohorts.

Clinical Variable	Statin use (*n* = 384)	No statin use (*n* = 384)	*p* value
Length of hospital stay (days)	6.0 (3.0–11.0)	6.0 (4.0–12.0)	0.27
Mechanical ventilation n (%)	77 (20.0%)	93 (24.2%)	0.07
Days on ventilator	13.5 (3.2–31.0)	12.8 (2.0–34.1)	0.77
Mortality after hospitalization n (%)	66 (17.1%)	119 (31%)	<0.001
Haemodialysis n (%)	21 (5.4%)	27 (7.0%)	0.41

### Clinical Outcomes of Propensity-Matched Cohort

Comparison and variation in clinical outcomes in the propensity-matched cohort between statin and non-statin use are presented in Table 4. The mortality in statin users was 17.1% compared to 31% in non-statin users.

### Multivariable Adjusted Overall Cohorts

The clinical outcome in the multivariable-adjusted overall cohort [odds ratio (OR) 0.55, 95% confidence interval (CI) 0.37-0.63] revealed that statin users had a significant effect on the primary endpoint (mortality during hospitalization). The odds ratio was defined as “a measure of association between the statin use/exposure and the clinical outcome.” The secondary endpoint was attained in 77 (20%) patients receiving statins compared to 93 (24.2%) non-statin users. Statin users also showed reduced odds in the multivariable-overall (OR 0.76, 95%CI 0.58-1.00), but this was statistically not significant (Table 5).

**TABLE 5:** Clinical outcome in multivariable adjusted cohorts and propensity matched cohorts.

	OR*	95% CI	*p*-value
Primary endpoint (mortality during hospital stay)			
PS-matched	0.52	0.33-0.6 4	<0.001
Multivariable (PS-matched)	0.53	0.35-0.67	<0.001
Multivariable (overall)	0.55	0.37-0.63	
Secondary endpoint (invasive mechanical ventilation during hospital stay)			
PS-matched	0.80	0.64-1.02	<0.001
Multivariable (PS-matched)	0.89	0.68-1.20	<0.001
Multivariable (overall)	0.76	0.58-1.00	

PS, propensity score; OR, odds ratio; CI, confidence interval. * Odds ratio (OR) was defined as “a measure of association between the statin use/exposure and the clinical outcome”.

### Statistical Comparison Between at Risk, Death, and Discharge Status in the Propensity-Matched Cohort

**FIGURE 2:**
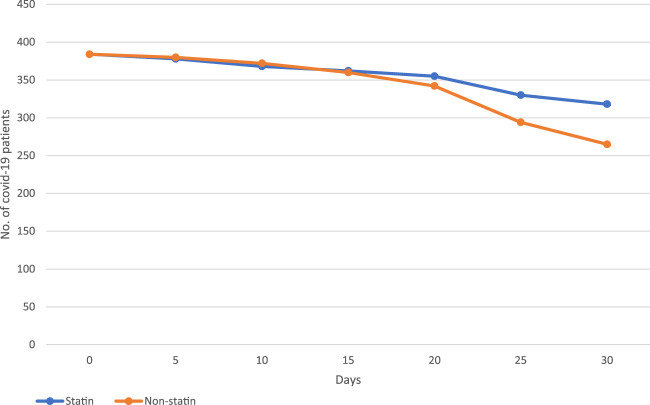
Survival curves depicting statin and non-statin use after Propensity score matched.

The hospitalized COVID-19 patients were followed over a 5-day interval for over 30 days (Figure 2). The incidence rate of mortality during the 30-day follow-up was 0.09 cases per 100-person-day in statin users (the mortality rate was 17.1%) in comparison to 0.11 cases per 100-person-day in non-statin users (the mortality rate was 31%) (Table 6). The matched statin users had more severe baseline clinical characteristics and higher proportions of pre-existing co-morbidities. We used the mixed-Cox model without involving the time-varying exposures in our propensity score matched (PSM) cohort; the statins significantly reduced the mortality incidence (aHR:0.25, 95% CI: 0.12-0.38, *p* = 0.001) (Table 6). The graph (Figure 2) shows the survival pattern among COVID-19 patients between statin and non-statin users. The “at-risk” COVID-19 cases were defined as the total number of patients minus deaths and the “survival” COVID-19 cases were defined as the total number of at-risk COVID-19 patients minus deaths.

**TABLE 6:** Incidence rate and hazard ratios to evaluate the association between in-hospital statin therapy and mortality.

Statin versus Non-statin^a^	Unmatched							Matched				
	Crude incidence			Cox model time-varying exposure		Marginal-structural model		Crude incidence after PSM			Mixed Cox model	
	IR	IRR (95%CI)	*p* value	aHR (95%CI)	p value^b^	aHR (95%CI)	*p* value	IR	IRR (95%CI)	*p* value	aHR (95%CI)	*p* value
	0.09 vs. 0.11	0.39 (0.21–0.57)	0.023	0.31 (0.14-0.48)^c^	0.0004	0.39 (0.25-0.53)^c^	0.017	0.08 vs. 0.14	0.23 (0.09–0.36)	<0.001	0.25 (0.12-0.38)^d^	0.001

a There were 524 and 1102 COVID-19 hospitalized patients in unmatched statin and non-statin groups respectively. After PSM with 1:1 ratio, there were 384 and 384 COVID-19 hospitalized patients in matched statin and non-statin groups, respectively.

b SPSS statistical analyses 2.0 was used to calculate the *p* values.

c The hazard ratio was adjusted for age, gender, BMI, co-morbidities (HTN, CAD, CLD, CKD, CVA, and heart failure), laboratory values (WBC count, D-dimer, CRP, ESR and SpO2), mechanical ventilation, hemodialysis, and duration of hospitalization.

d aHR was calculated based on mixed-effect Cox model with adjustment of age, gender, CAD, increase D-dimer, increase CRP, increase ESR at admission.

IR, Incidence rate; IRR, Incidence rate ratio; aHR, adjusted hazard ratio.

### Statin Frequency, Regimens, and Dosage

**FIGURE 3:**
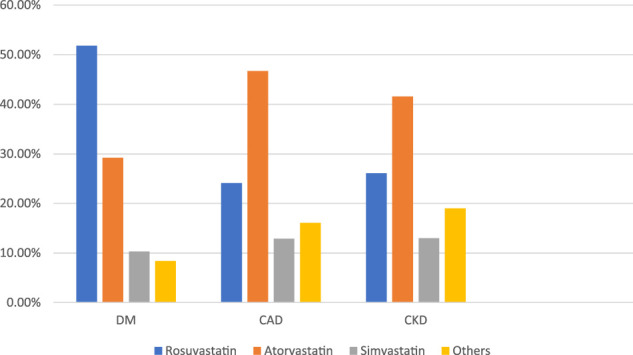
Preferred statin in COVID-19 patients.

In our study, physicians prescribed Rosuvastatin, the most preferred statin, to COVID-19 patients (Figure 3). It was observed that Rosuvastatin was most effective than the other statin group in reducing LDL, triglycerides, and total cholesterol and increasing HDL levels. As presented in Table 7, in COVID-19 patients with co-morbidities, Rosuvastatin was the most preferred statin to be prescribed by the physicians in COVID-19 patients with diabetes mellitus (51.8%). In comparison, atorvastatin was the most preferred statin in CAD and CKD patients (46.7 and 41.6%, respectively). The highest tolerable dose for Rosuvastatin was 40 mg, and for atorvastatin and simvastatin, it was 80mg, respectively. However, Rosuvastatin was prescribed at a 10 mg daily dose, whereas atorvastatin and simvastatin were prescribed at a 20 mg dose for 6 weeks.

**TABLE 7:** Preferred statin in a COVID-19 patient with co-morbidity.

COVID-19 patients with co-morbidities	Rosuvastatin	Atorvastatin	Simvastatin	Others	Total
Diabetes mellitus	110 (51.8%)	62 (29.2%)	22 (10.3%)	18 (8.4%)	212
CAD	15 (24.1%)	29 (46.7%)	8 (12.9%)	10 (16.1%)	62
CKD	22 (26.1%)	35 (41.6%)	11 (13%)	16 (19%)	84

CAD, coronary artery disease; CKD, chronic kidney disease.

## Discussion

This cohort study evaluated the effect and outcome of statin use (antecedent and in-hospital) on patients’ clinical manifestation, laboratory results, and clinical outcome. The principal findings in this analysis show that: 1. COVID-19 patients have commonly used statins (32.2%) prior to hospital admission due to their impending co-morbidities (hypertension, diabetes mellitus, coronary artery disease), 2. COVID-19 patients were older males, with a higher BMI, 3. the total lipid profile, WBC count, and C-reactive protein, were more favorable in statin users, and 4. the clinical outcome was significantly better in statin users.

Several literature studies have postulated the role of statins in COVID-19 patients (Hope et al., 2019; Fan et al., 2020; Pal et al., 2021; Wu et al., 2021). In our study, the predominance of COVID-19 in the elderly population can be elucidated for two reasons. One, the elderly population is more likely to be associated with co-morbidities such as hypertension, diabetes mellitus, and coronary artery disease, in contrast to the younger and middle age group. Two, the elderly population is more immune-compromised. These factors form precursor mediums in developing inflammatory-triggered cytokine storm syndrome, which further complicates the host defense mechanism and increases the probability of hospitalization, as observed in our study. The prevalence of hypertension in our hospitalized COVID-19 patient cohort was 75%. The proposed pathogenesis for this common coexistence is elevated levels of IL-6, tumor necrosis factor *α* (TNF α), and granulocyte-macrophage colony-stimulating factor resulting in COVID-19 induced cytokine storm. The cytokine storm further induces an imbalance in the renin-angiotensin system (RAS) and the NADH/NADPH oxidase causing aggravated pulmonary damage (Mahmudpour et al., 2020). The resulting underlying pulmonary pathology triggers the occurrence of respiratory symptoms, as observed in our study.

The higher percentage of diabetes mellitus in our hospitalized COVID-19 patients is elucidated by the relationship between elevated glucose levels and SARS-CoV-2 replication. The enhanced viral replication seen in COVID-19 diabetic patients is due to sustained glycolysis, increased mitochondrial reactive oxygen species production, and activation of hypoxia-inducible factor 1 *α*. Our study shows that COVID-19 patients with diabetes mellitus are at a higher risk for hospitalization and have a higher mortality rate due to dysregulated immune response (Hammad et al., 2019). Other causes described in the literature for higher prevalence of diabetes in COVID-19 patients are reduced macrophage function, decreased mobilization of polymorphonuclear leukocytes, and inhibition of tumor necrosis alpha activity on T-cells (Galicia-Garcia et al., 2020; Mahmudpour et al., 2020). Statins inhibit the major protease (Mpro) and RNA-dependent RNA-polymerase (RdRp), thereby lowering serum IL-6 and modulating macrophage activity (Coperchini et al., 2020; Costela-Ruiz et al., 2020; Pawlos et al., 2021). These actions provide an ameliorated immune environment and reduce the severity of COVID-19 illness, further reflected by laboratory inflammatory markers (CRP, WBC count) as observed in our study (Ponti et al., 2020).

COVID-19 also triggers the risk of coronary vascular disease in patients with hypercholesterolemia and atherosclerosis (Grzegorowska and Lorkowski, 2020; Nishiga et al., 2020; Basu-Ray et al., 2021). Statins generally lower LDL-cholesterol by inhibiting the HMG-CoA reductase enzymes in the liver, further inhibiting HMG-CoA conversion into mevalonate and reducing the total cholesterol (Feingold et al., 2000). recent studies at the molecular level have shown that statins suppress TLR4/MyD88/NF kB signaling (Senol et al., 2021). These molecular level changes make the patient move into a protective anti-inflammatory state. The role of an intracellular inflammasome NLRP3 (NOD-, LRR- and pyrin domain-containing protein 3) is also well established. This intracellular sensor is enabled by oxidized LDL and TNF α, causing activation and release of cytokines in patients with metabolic dysfunction, resulting in cardiovascular complications in COVID-19 patients. Statins inhibit NLRP3 inflammasome activation by suppressing the oxidized LDL, and TNF α, and improving the cardiovascular functional outcomes in COVID-19 patients (Umakanthan et al., 2021b; Koushki et al., 2021). These beneficial effects of statins on cholesterol levels have been observed in our lipid profile of matched cohort patients.

The role of COVID-19 in causing endothelial dysfunction is well established (Gavriilaki et al., 2020; Bonaventura et al., 2021). This effect is mainly attributed to its microvasculature immune-inflammatory process, reflected by higher laboratory D-dimer levels (Gencer et al., 2020). Higher D-dimer levels impart the extent of thrombus formation and predict the risk of thrombo-embolic complications (Al-Ani et al., 2020). Statins improve endothelial functions by enervating TGF ß, VEGF and reducing serum PAI-1 levels (Tsujinaka et al., 2017). They exert their anti-thrombotic effects by reducing the tissue factor expression and downregulating the blood coagulation cascade. This endothelial function enhancing property of statins has been reflected in many studies by the lower D-dimer levels in COVID-19 statin users, however in our study, the D-dimer levels for statin users vs. non-statin users were not significantly different (*p* = 0.37). (Vuorio and Kovanen, 2020; Ferrari et al., 2021).

The relation between hypertriglyceridemia and the risk of developing atherosclerosis has been contrasting. The initial studies revealed that patients with severe hypertriglyceridemia had a higher risk of developing atherosclerosis. However, the recent data suggest that patients with severe hypertriglyceridemia have a large-sized lipoprotein, hence restricting its entry into the arterial intima (Nelson, 2013; Prasad et al., 2019). Thus, based on this observation in our study, patients with moderate elevations of triglyceride are at higher risk of developing atherosclerosis. In our matched cohort, statin showed a more successful cholesterol-lowering effect (higher HDL and lower triglyceride) in patients with hypertriglyceridemia than in patients without elevated triglyceride levels.

This observational study aims to estimate the effects of statins on the treatment outcome of hospitalized COVID-19 patients. Our study’s selection of patients on statins was influenced by subjective characteristics (e.g., co-morbidities). Due to the subjective selection, the statin-treated patients’ baseline characteristics differed from the non-statin patients. The logistic regression model determined these systemic differences in the baseline covariates and the confounding factor-induced biased results between statin users and non-statin users. In our study results, rosuvastatin was the most preferred statin to be prescribed by the clinician. In a meta-analysis performed by Weng et al., they discussed that rosuvastatin (at 10 mg) and atorvastatin (at 20 mg) were the two most effective statins that could reduce LDL and raise HDL levels (Weng et al., 2010). The STELLAR trial and the PULSAR study demonstrated a remarkable reduction in LDL, total cholesterol, and triglycerides compared to other statins (Clearfield et al., 2006; Welty et al., 2016). Many patients with a high risk of coronary vascular disease generally fail to attain the recommended LDL goal. However, the PULSAR trial demonstrated that with rosuvastatin at 10 mg, many patients could attain the LDL goal of less than 100 mg/dl. These patients had either atherosclerosis, type 2 diabetes mellitus, or were at elevated risk of coronary vascular disease (Clearfield et al., 2006).

Numerous studies have favored the use of statins in COVID-19 patients (Fan et al., 2020; Zhang et al., 2020; Wu et al., 2021). The National Health Institute, United States has recommended that COVID-19 patients continue statin therapy to prevent and treat cardiovascular disease (Vuorio and Kovanen, 2020). This safe and cost-effective drug has been proven beneficial during hospitalization and linked with better clinical outcome (Zhang et al., 2020). The diagnosis of COVID-19 in adult patients with cardiovascular disease has triggered clinicians to initiate statin therapy (Madjid et al., 2020).

The endothelial dysfunction caused by COVID-19 is well combatted by statin-induced improved endothelial function, thereby reducing the risk of thrombotic complications (Gavriilaki et al., 2020). Retrospective meta-analyses and cohort studies have shown better clinical outcomes in COVID-19 patients with statin use (Fan et al., 2020; Zhang et al., 2020; Wu et al., 2021). This includes investigating such patients for the severity of the infection and in-hospital mortality. However, such results need a cautious interpretation since most statin users are old males with hypertension, diabetes mellitus, and cardiovascular disease; these are proven factors to exacerbate COVID-19 prognosis (Scheen, 2021). The effects of these confounding factors need to be eliminated by rigorous statistical analyses as done in our study (Arbogast et al., 2013). Statins modulate and modify ACE2 levels and produce epigenetic modifications, thereby preventing the progression to Acute Respiratory Distress Syndrome (ARDS) and also limit the severity of COVID-19, as shown in COVID-19 literature studies (Castiglione et al., 2020; Pawlos et al., 2021).

Our multi-centric study analyses during the COVID-19 pandemic have demonstrated that prior statin use significantly reduces hospital mortality rates. The literature studies on the effect of statins on hospitalized COVID-19 patients validate the results presented in our manuscript. A study done by Zhang et al. showed some variations in their results compared to our study due to the differences in the study size (<10% of hospitalized patients were statin users) and the genetic form of the Chinese population (Zhang et al., 2020). Most studies originated from China, and few recent meta-analyses showed the reflection of statin effects in the European and North American patient population. In a study conducted in the US, statin use showed a remarkable reduction in mortality rates in hospitalized COVID-19 patients, as displayed in our cohort study (Kow and Hasan, 2020; Wu et al., 2021). This US study compared the demographic, clinical, and laboratory variables between statin users and non-users by implementing the student t-test. The hospital discharge events have competed with in-hospital mortality using competing events analysis. Confounding bias was reduced by applying a multivariable regression model and propensity score analysis to conclude the inverse relationship between statin use and mortality rates. However, the findings and results varied in study size, and type of statin regimens (Saeed et al., 2020).

### Limitations of the Study

Our study was conducted from the time interval of 1 June 2020, to 30 April 2021. Since Genesequencing was not available in these three multispecialty hospitals where the study was conducted, we could not incorporate the details of our study’s gene sequencing and variants results. Furthermore, the samples from COVID-19-positive patients had to be sent to regional gene sequencing laboratories, which would require ethical clearance from the National Regional Research Centre for the results obtained to be used in our study. Since intra-mural or extra-mural grants did not fund our study, we could not afford to bear the enormous financial burden for this aspect.

The detailed age-wise separation was not reflected in detail in our manuscript as the other age groups were not statistically significant to provide a positive impact through a graphical representation using plot analysis by the statistical analyzer software as used in our study (both joint-plot and Forest plot analysis were attempted for this purpose). Hence, we decided only to present the age range that positively impacted our study, as shown above.

## Conclusion

This is the first multi-centric cohort study conducted in India during the worst wave of the COVID-19 pandemic. We initially involved 1626 COVID-19 patients, followed by a 1:1 propensity-score matching, and eventually, 768 patients were included. After adopting appropriate statistical methods to minimize confounding, we observed that statins users had favorable laboratory findings and clinical outcomes compared to non-statin users. These findings may further strengthen the focus on the beneficial effects of administering statins in COVID-19 patients.

## Data Availability

The original contributions presented in the study are included in the article/supplementary material, further inquiries can be directed to the corresponding author.
